# Is Neuromorphic MNIST Neuromorphic? Analyzing the Discriminative Power of Neuromorphic Datasets in the Time Domain

**DOI:** 10.3389/fnins.2021.608567

**Published:** 2021-03-25

**Authors:** Laxmi R. Iyer, Yansong Chua, Haizhou Li

**Affiliations:** ^1^Neuromorphic Computing, Institute of Infocomms Research, A*Star, Singapore, Singapore; ^2^Huawei Technologies Co., Ltd., Shenzhen, China; ^3^Data Center Technologies Lab, Department of Electrical and Computer Engineering, National University of Singapore, Singapore, Singapore

**Keywords:** spiking neural network, spike timing dependent plasticity, N-MNIST dataset, neuromorphic benchmark, spike time coding

## Abstract

A major characteristic of spiking neural networks (SNNs) over conventional artificial neural networks (ANNs) is their ability to spike, enabling them to use spike timing for coding and efficient computing. In this paper, we assess if neuromorphic datasets recorded from static images are able to evaluate the ability of SNNs to use spike timings in their calculations. We have analyzed N-MNIST, N-Caltech101 and DvsGesture along these lines, but focus our study on N-MNIST. First we evaluate if additional information is encoded in the time domain in a neuromorphic dataset. We show that an ANN trained with backpropagation on frame-based versions of N-MNIST and N-Caltech101 images achieve 99.23 and 78.01% accuracy. These are comparable to the state of the art—showing that an algorithm that purely works on spatial data can classify these datasets. Second we compare N-MNIST and DvsGesture on two STDP algorithms, RD-STDP, that can classify only spatial data, and STDP-tempotron that classifies spatiotemporal data. We demonstrate that RD-STDP performs very well on N-MNIST, while STDP-tempotron performs better on DvsGesture. Since DvsGesture has a temporal dimension, it requires STDP-tempotron, while N-MNIST can be adequately classified by an algorithm that works on spatial data alone. This shows that precise spike timings are not important in N-MNIST. N-MNIST does not, therefore, highlight the ability of SNNs to classify temporal data. The conclusions of this paper open the question—what dataset can evaluate SNN ability to classify temporal data?

## 1. Introduction

The remarkable performance and efficiency of the brain have prompted scientists to build systems that mimic it—for studying biological function as well as improving engineering systems. Early neural networks, networks of the first and second generations do not have neurons that spike. These networks, known as artificial neural networks (ANNs) have real-valued outputs and can be seen as time averaged firing rates of neurons. The networks of the third generation (Maass, 1997; Vreeken, 2003), known as spiking neural networks (SNN) explicitly employ spikes as their mechanism for computation. Third generation networks are more mathematically accurate models of biological neurons. A neuron of the third generation network receives incoming spikes through its synapses and fires a spike when its membrane potential exceeds a threshold. Such a neuron can use spike time coding, described below. Before we describe spike time coding, we will first enumerate the different definitions of firing rate currently used.

**Figure 1:**
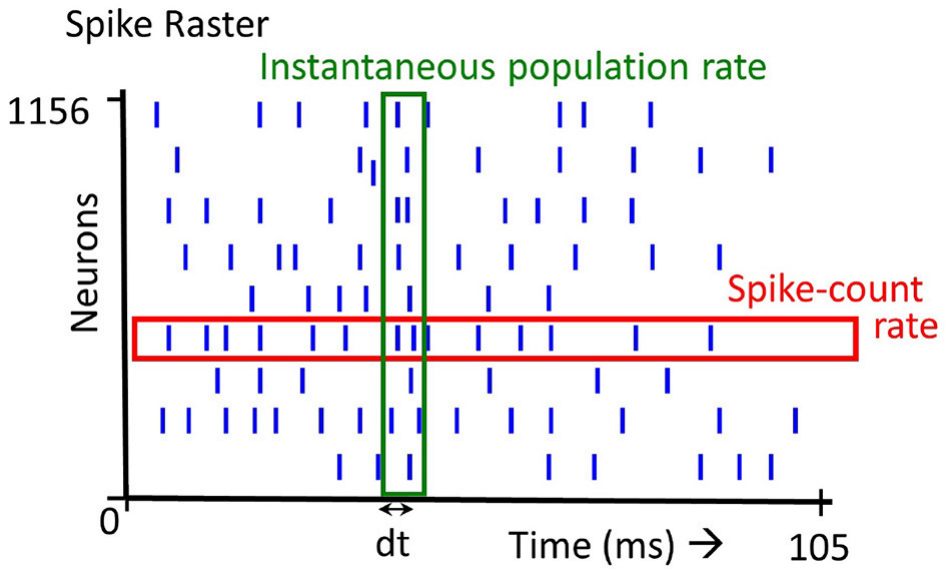
Spike rate definition: there are several definitions of spike rate. Averaging over all the spikes emitted by a single neuron (spikes in the red box), we get the *Spike-count rate*. Averaging over the spikes emitted at a time instant by all the neurons (spikes in the green box), we get the *Instantaneous population rate*.

The firing rate of a spiking neuron is defined in several ways: (1) The time averaged firing rate is the number of spikes fired by a neuron over a certain duration, (2) The instantaneous population firing rate is the number of spikes elicited by a population of neurons in a small time window, (3) The trial averaged firing rate of a neuron firing is the average number of spikes across trials. Note that definition (2) and (3) denote firing rate as a variable in time. The first two definitions are illustrated in Figure 1. In this paper, we focus primarily on the first definition (Figure 1, spike-count rate) but also consider the second definition (Figure 1, instantaneous population rate).

Scientists have debated over how neurons code information—whether the brain follows a rate code or a temporal code (Brette, 2015). Rate code makes use of the firing rate of neurons while temporal code makes use of the precise spike timing of the neurons. The issue of time and rate coding, as summarized in Brette (2015) is as follows: does spike firing rate of a neuron capture most of the important information and computations, rendering the exact timing of spikes unnecessary?

Several studies have highlighted the importance of precise spike times. Firstly, Gerstner et al. (1999) shows that there are specialized subsystems for which the precise timing of spikes are relevant. The specialized subsystems include the electrosensory system of electric fish (Heiligenberg, 1991; Metzen et al., 2016) and the auditory system of barn owls (Carr and Konishi, 1990; Konishi, 1993; Gerstner et al., 1999; Wagner et al., 2005; Keller and Takahashi, 2015; Carr et al., 2016). Behavioral experiments on owls show that they can locate sound sources in complete darkness with extreme precision. They can detect a temporal difference of around 5μ*s* between the left and right ear. Such precise calculations invalidate the use of an averaging mechanism in the brain. Secondly, Thorpe et al. (2001) details several arguments for spike time codes. Experiments show that primates are able to perform visual classification as fast as 100–150 *ms* after the stimulus is presented (Thorpe et al., 2001; Kirchner and Thorpe, 2006; Butts et al., 2007; Crouzet et al., 2010). Given that this information must have passed about 10 layers of processing, each individual processing stage is completed on average in only 10 *ms*, rendering a time averaged rate coding mechanism highly unlikely (Thorpe et al., 2001; Butts et al., 2007). Further, the number of photoreceptors that are present in the retina and the resolution of the images processed invalidate an instantaneous population rate code (Thorpe et al., 2001).

Spike time coding does not need a large number of spikes or many neurons to quantify large values, but can do so by varying the spike timing of a few neurons. As a result, spike time codes allow more efficient computation. If a SNN is just using time-averaged or instantaneous population rate codes, it would be less efficient than ANNs, as it would need to run for long periods of time or employ many neurons to compute accurate averages of spike rates. The main advantage of the SNNs over the previous two generations of neural networks is that they can, in principle, employ spike time coding for higher efficiency.

Neuromorphic engineering incorporates hardware and software systems that mimic architectures present in the nervous system. An important aspect of neuromorphic engineering is that it attempts to utilize the computations of biological neurons, circuits and architectures and use them in learning and information processing. The neural networks most closely related to biological neurons and still widely used in engineering are spiking neural networks (SNN). Neuromorphic engineering is a multidisciplinary field that involves computer science, biology, physics, mathematics, and electronics engineering.

SNNs and their class of learning algorithms form a substantial but not all of learning algorithms in the neuromorphic community. Indeed, our community should not just draw ideas from neuroscience, but could benefit much from ideas in the more general machine learning or AI community. Hence, the boundary between neuromorphic and deep learning, if there was even a clear one to begin with, is now even less so. Hence, in this paper, we use neuromorphic algorithms/systems in its more narrow sense to refer to spiking neural networks. This is for several reasons. Most major neuromorphic systems use SNNs as their software^1^ (Benjamin et al., 2014; Merolla et al., 2014). Further, the only distinguishing factor that separates neural networks generally used in neuromorphic architectures from neural networks in machine learning is that the former are spiking. Precise spike timing has been perceived by many in the community as an important source of temporal information, which drives the many on-going studies in SNN/neuromorphic learning algorithms and neuromorphic datasets. We also refer to neuromorphic datasets derived from static images as neuromorphic datasets in the paper.

Along with the advances of neuromorphic engineering, there arises the need for a neuromorphic dataset to benchmark different SNNs. In Computer Vision, MNIST (LeCun et al., 1998) and Caltech101 (Fei-Fei et al., 2004) are examples of well known image datasets. MNIST is a dataset of handwritten digits and consists of 60,000 training patterns and 10,000 test patterns. Caltech101 has pictures of objects belonging to 101 categories. Categories in Caltech101 have 40–800 images, with most categories having around 50 images. Recently neuromorphic algorithms have been tested against MNIST (e.g., Querlioz et al., 2013; Diehl and Cook, 2015; Kheradpisheh et al., 2018; Mozafari et al., 2018; Thiele et al., 2018). To do this, images are converted to spikes using different methods. For e.g., Querlioz et al. (2013) and Diehl and Cook (2015) convert images to Poisson spike trains with spike rates proportional to the intensity of the pixels. (Thiele et al., 2018) draw their mean spike rate from a Gaussian distribution with spike rates proportional to the intensity of the pixels. Kheradpisheh et al. (2018) and Mozafari et al. (2018) convert images to spikes with spike times inversely proportional to image contrast. However, to advance the field of neuromorphic algorithms, a dataset whereby features are encoded in asynchronously in time is required, which incidentally renders any data pre-processing unnecessary. N-MNIST, N-Caltech101 (Orchard et al., 2015a), MNIST-DVS and CIFAR10-DVS are datasets recorded by moving either an event-based vision sensor or the image from a pre-existing Computer Vision dataset and recording the resultant images. For example, Neuromorphic MNIST (N-MNIST) and Neuromorphic Caltech101 (N-Caltech101) (Orchard et al., 2015a) are recorded by moving an ATIS vision sensor (Posch et al., 2011) across the original MNIST and Caltech101 patterns respectively in 3 predefined directions. The ATIS vision sensor is a neuromorphic sensor that records pixel-level intensity changes in the scene, based on the principles of the retina. The N-MNIST and N-Caltech101 patterns are therefore, represented as events occurring at pixel locations. The N-MNIST dataset has been successfully tested on many recent neuromorphic algorithms (for e.g., Lee et al., 2016, 2020; Thiele et al., 2018; Wu et al., 2018; Jin et al., 2019; Kim et al., 2020). Image-derived neuromorphic datasets are just but a subset of all neuromorphic datasets. An example of a dataset that is not derived from images is DvsGesture (Amir et al., 2017), which is recorded from hand and arm movements. DvsGesture is a dataset consisting 1,342 hand and arm movements from 29 subjects and 11 gestures.

To summarize the above, there are currently several examples of DVS-based datasets which are useful for benchmarking SNNs. In general, spikes can encode information in two ways: (1) Their precise spike timings (although spikes may be subject to jitter and an SNN should be able to learn these as well) and (2) Firing rate or spike counts over a relatively large time window.

Given the properties of SNN, we would like to further understand how it can learn information encoded in precise spike timing (over various time scales), and not just simply spike counts in a certain time window. At same time, we hope to see more of DVS or other event-based sensor generated datasets, as these are naturally compatible with SNNs. As such, we hope to see more event-based datasets that encode information in precise spike timing on top of spatially encoded information and rate-coded/spike count encoded information, so as to improve/benchmark SNN learning capabilities. Precise timing of spikes is an important aspect of SNNs, and there is ample evidence in the brain that precise timing of spikes can be constructively used in spike-based calculations to increase efficiency.

In addition to enabling spike timings in their calculations, SNNs have other benefits—for example, SNNs enable low power computation, due to the sparse computation and binary nature of the output, and we agree that datasets without information encoded in spike timing can be used to assess such capabilities. If datasets such as N-MNIST were used predominantly to assess such capabilities, it may not matter whether they have information coded in the timing of spikes necessary to classify the dataset. N-MNIST and other datasets generated from static images, are implicitly regarded as having both spatial and temporal information, and widely and generically used as such (for e.g., Thiele et al., 2018; Wu et al., 2018; Jin et al., 2019; Cheng et al., 2020; Kim et al., 2020). Therefore it becomes extremely important to understand whether such temporal information encoded in spike timing information is actually present, necessitating a study such as ours.

Orchard et al. (2015a) mentions that in N-MNIST and N-Caltech101, the movement of the ATIS sensor mimics retinal saccades. However, our visual system is designed to extract information about the 3D world from many 2D image projections formed by the retina (Elder et al., 2016). Visual information is integrated across retinal saccades (Fiser and Aslin, 2002) to provide a more holistic visual representation, for example to group visual input to separate image from ground (Blake and Lee, 2005). In addition, as George (2008) describes, we are very adept at recognizing images despite different rotations, scales, and lighting conditions (also Simoncelli, 2003). Such an integrated representation of objects is obtained from data varying continuously in time over all these different dimensions, in ways that conform to laws of physics (Blake and Lee, 2005; George, 2008; Mazzoni et al., 2011; Lake et al., 2016; Keitel et al., 2017). Therefore, time is probably acting as a supervisor providing useful information to enable us to create such a holistic representation (George, 2008). It is therefore necessary to ask if saccadic movements of the camera used to record N-MNIST and N-Caltech101 gather information that is just as rich and critical for classification. Saccades in these datasets are constructed by moving a camera over 2D static images in a predefined manner. This may not match the description of retinal saccades given by Fiser and Aslin (2002) and George (2008). At the very least it should provide additional information from the original MNIST and Caltech101. We therefore want to know what role time plays in these datasets. We commence our study with both N-MNIST, N-Caltech101, and DvsGesture but focus the rest of this study on N-MNIST alone.

In this paper, we ask two questions about neuromorphic datasets recorded from pre-existing Computer Vision datasets by moving the images or a vision sensor:

1. These datasets are encoded in a spatio-temporal domain. Does the timing of spikes in these neuromorphic datasets provide any useful information?
2. Do these neuromorphic datasets highlight the strength of SNNs in classifying temporal information present in the precise spike timings?

The second question has two parts. The strength of an SNN algorithm in classifying information encoded in spike timing is highlighted if: (1) the neuromorphic dataset has information coded in precise spike timings that can be potentially utilized by the SNN, and (2) The SNN is able to utilize this temporal information effectively. An important and related question is if the current SNNs are able to exploit spike timing information. It is important that the neuromorphic datasets that are used have information in spike timings that can then be potentially exploited by SNNs for classification.

The above two questions are important from various viewpoints—from a general machine learning perspective, we want to know if these neuromorphic datasets can be classified by ANNs just as well, or even more efficiently. From the neuromorphic perspective, a neuromorphic dataset should be able to highlight the unique properties and strengths of SNNs over ANNs in certain machine learning tasks. From the neuroscience point of view, it would be interesting to investigate if this method of recording from static images would gather additional information in the time domain than that available in the original Computer Vision datasets (such as MNIST and Caltech101), which can then be further utilized by some learning algorithms.

To address the questions above, we present several experiments with the neuromorphic datasets. A list of all the experiments and the datasets used are given in Table 1. While we want to assess neuromorphic datasets derived from static images, we focus on N-MNIST in this paper. We do the initial experiment (see section 3) on both N-Caltech101 and N-MNIST to show that the same trend holds for both datasets. In the experiments with ANN (see section 3) and the DSE experiments (see section 5), we use DvsGesture as an example of a dataset derived from hand movements instead of static images—to contrast against N-MNIST (and N-Caltech in section 3).

1. Our paper only applies to neuromorphic datasets derived from static images by use of a vision sensor (such as DVS or ATIS, Lichtsteiner et al., 2008; Posch et al., 2011; Brandli et al., 2014). In order to compare them with a neuromorphic dataset that is not derived from static images, we present experiments on the DvsGesture dataset.
2. By information in the time domain or temporal information, we specifically refer to spike timing, and all its derivatives, such as difference in spike timings, such as inter-spike intervals (ISI) and spike timing sequences across a population.

**Table 1:** A summary of the experiments.

**No**.	**Algorithm**	**Datasets**	**Experiment**
1.	ANN	N-MNIST, N-Caltech101, DvsGesture	All spikes are summed up over time (thereby eradicating spike time information), and classified by an ANN. This is to examine if the lack of spike timing information affects accuracy.
2.	RD-STDP and STDP-tempotron	N-MNIST and DvsGesture	Two algorithms are compared, one which classifies static data, and another that can classify spatio-temporal data, on the two datasets.
3.	RD-STDP	N-MNIST	We explore if fixing the output spike time affects the accuracy.
4.	Population rate dependent plasticity (new rule)	N-MNIST	A handcrafted plasticity rule based on the population rate is used to classify the dataset—to understand if a purely population rate based rule can affect the accuracy.

Our empirical study contains two parts—first is to examine the classification of neuromorphic datasets using ANNs. We compare ANNs, which do not use temporal information for classifications, with state-of-the-art SNNs. The second part of our paper has several experiments using SNNs with spike timing dependent plasticity (STDP). The purpose of the second part is to examine if additional information is encoded in the timing of spikes.

For SNN experiments, we chose spike-timing dependent plasticity (STDP) as firstly, the learning rule is based on the precise timing of spikes, and secondly, by relaxing the time constants of the synaptic traces, STDP becomes less sensitive to spike timing and approximates a rate-based learning rule. This property can then be exploited in an empirical study of the usefulness of time domain information encoded in any spatio-temporal dataset.

We start off with a description of N-MNIST, N-Caltech101, and DvsGesture datasets after which we describe our first experiment. Here, N-MNIST, N-Caltech101, and DvsGesture are time-collapsed into static images, by summing the number of spikes over time. These time-collapsed images are trained on an ANN. We then describe a design space that further experiments would explore, followed by other experiments that compare the performance of temporal and rate based SNNs on the N-MNIST dataset. This is followed by an experiment that classifies the N-MNIST dataset using an SNN trained with a data-derived STDP rule based on instantaneous population rates. Finally, we conclude with a discussion on the implications of these results, and other related questions. All accuracies reported in this paper are based on the test sets.

## 2. N-MNIST and N-Caltech101 Data Format

The N-MNIST dataset is created by moving the ATIS vision sensor over each MNIST image. This is done for all 60,000 training images and 10,000 test images in MNIST. The camera has 3 pre-defined movements (or *saccades*). Each N-MNIST spike train is 360*ms* long—divided into 3 saccades. The first saccade occurs during the first 105 ms (0–105 ms), the second saccade in the next 105 ms (105–210 ms), and the third saccade in the next 105*ms* (210-315 *ms*)^2^ (Cohen et al., 2016). Finally there is a 45 ms additional time appended to end of 315ms to ensure that the last events have an effect on learning (Cohen et al., 2016).

N-MNIST patterns are represented as *events*, each occurring at a specific pixel location or *address* at a particular time (each event has a time stamp in μs). This is known as the *address-event representation (AER)* protocol. Events elicited due to an increase in pixel intensity are characterized as *ON* events, and decrease in pixel intensity, as *OFF* events.

In our experiments we consider *ON* events in the first saccade (0–105 *ms*) for most experiments. We reduce the time resolution of the spike trains by binning events with μs time stamp into ms intervals. For the first two important experiments (sections 3, 5), we examine N-MNIST with all saccades as well, and do not observe a significant change in performance.

Caltech101 contains 8709 images, and N-Caltech101 is created in the same manner from the ATIS vision sensors.

DvsGesture is comprised of 1,342 patterns. A set of 29 subjects stood against a stationary background and performed 11 hand and arm gestures each with 3 illumination conditions. These gestures were recorded using the DVS128 (Jimenez-Fernandez et al., 2010) camera. In contrast to the previous datasets, this dataset is not derived from static images, but from dynamic movement. 11 classes correspond to gestures such as *hand waving, arm rotations clockwise, arm rotations counter-clockwise*, and *clapping*. The 11*th* class, *Other* consists of a gesture invented by the subject. For ease of classification, we took out the *Other* class.

## 3. Experiment: Training N-MNIST, N-Caltech101, and DvsGesture Images With an Artificial Neural Network

This experiment examines the performance of frame based versions of N-MNIST, N-Caltech101 and DvsGesture on artificial neural networks (ANN). Each frame is created by summing the number of events over time—we henceforth refer to these frames as *time-collapsed* images. We want to compare the performance of neuromorphic datasets derived from static images (i.e., N-MNIST and N-Caltech101) to a dataset recorded from real-time movements, i.e., DvsGesture dataset.

**Figure 2:**
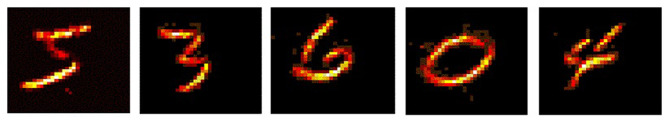
N-MNIST time collapsed images: N-MNIST patterns are collapsed in the time dimension to static images with pixel intensity proportional to the spike rate of the pixel. These images are trained on an ANN to examine how the removal of the temporal component in N-MNIST affects the performance. The above are 6 such images created from N-MNIST time-collapsed patterns.

In this experiment, N-MNIST, N-Caltech101, and DvsGesture patterns are collapsed in the time dimension to static images with pixel intensity proportional to the spike rate of the pixel (see Figure 2 for examples of collapsed images). The conversion from AER to static images is done as follows. Each pattern *p* can be represented as a set of spike trains, one for each pixel. The spike train for pattern *p*, pixel *x* is $s^{x,p}=\left\{t_{1}^{x,p},t_{2}^{x,p},...t_{n}^{x,p}\right\}$ where each element denotes the time of spike. Note that $t_{1}^{x,p},...,t_{n}^{x,p}$ are in the range [0, 105]*ms* since we consider only saccade 1 (*ON* polarity). The normalized spike counts *C*^*x,p*^ are calculated as follows:

(1) $$C^{x,p}=\frac{\sum_{i}^{n}g(t_{i}^{x,p})}{\max_{y}\sum_{i}^{n}g(t_{i}^{y,p})}\;$$

where the function *g*(*t*) is calculated as follows:

(2) $$g(t)=\{\begin{array}{ll} 1, & 0\leq t\leq 105ms;\\ 0, & \text{otherwise}. \end{array}$$

So *C*^*x,p*^ is the count of spikes, normalized by the highest spike count per pixel in pattern *p*. Note that spike counts are normalized per pattern, so patterns with low spike rates have their overall *C*^*p*^, i.e., normalized spike count vector for a pattern, increased.

Each *time collapsed* N-MNIST image pattern *p* is a 34 × 34 image with intensity values at each pixel *x* being *C*^*x,p*^ (see Figure 2 for a few examples of images). The patterns are trained in Keras on a CNN whose specifications are given in Table 2. The loss function used is cross entropy, and the Adadelta optimizer is applied^3^. After running 100 epochs, we get a test accuracy of 99.23%. We compare this to the performance of other state-of-the-art algorithms on N-MNIST in Table 3.

**Table 2:** Description of the CNN used for classifying N-MNIST and DvsGesture.

**Layer**	**Specification**
Conv2d	32 filters of size 3 × 3, ReLU activation
Conv2d	32 filters of size 3 × 3, ReLU activation
MaxPool2d	Size—2 × 2
Dropout	Rate—0.25
Conv2d	64 filters of size 3 × 3, ReLU activation
Conv2d	64 filters of size 3 × 3, ReLU activation
MaxPool2d	Size—2 × 2
Dropout	Rate—0.25
Fully connected	128 output neurons
Dropout	Rate—0.5
Fully connected	10 output neurons, softmax activation

**Table 3:** This table shows the accuracy of N-MNIST on several state-of-the-art algorithms.

**Method**	**Accuracy (%)**
Lee et al.: Training SNN using backpropagation (Lee et al., 2016)	98.74
HATS (Sironi et al., 2018)	99.1
Active perception with DVS (Yousefzadeh et al., 2018)	98.8
Spatiotemporal backpropagation (Wu et al., 2018)	98.78
SLAYER (Shreshtha and Orchard, 2018a)	99.2
DECOLLE (Kaiser et al., 2020)	96
HM2-BP (Jin et al., 2019)	98.84
Spike based supervised gradient descent (Lee et al., 2020)	99.09
LISNN (Cheng et al., 2020)	99.45
Segmented probability-maximization (Liu et al., 2020)	96.3
Graph based object classification (Bi et al., 2019)	99.0
Learnable membrane time constants (Fang et al., 2020)	99.61
Collapsed images with ANN	99.23

*Clearly our method is among the state of the art*.

In order to ensure that the results we are getting is not due to the time window of 0–105 *ms*, we repeated the same experiment by collapsing the images and summing spikes up over all three saccades, i.e., having a time window of 0–315 *ms*. The experiment was identical to the previous one except the time window was changed to 0–315 *ms*. We obtained an accuracy of 99.18% showing that the good results are not dependent on the time window of collapsing the images.

N-Caltech101 has images of different sizes. Each *time collapsed* N-Caltech101 image pattern *p* is resized to a 224 × 224 image. Image resizing is performed using bilinear interpolation. These images are trained on a VGG-16 convolutional neural network pretrained on ImageNet. The methodology used for training is detailed in another paper by our group (Gopalakrishnan et al., 2018), where we examine N-Caltech101 more thoroughly. A comparison of N-MNIST and N-Caltech101 performance on several algorithms is given in Table 4. As can be seen, our method obtains close to state of the art accuracy with N-MNIST and N-Caltech101 datasets respectively.

**Table 4:** This table shows the accuracy of N-Caltech101 on several state-of-the-art algorithms.

**Method**	**Accuracy (%)**
HFirst (Orchard et al., 2015b)	5.4
HATS (Sironi et al., 2018)	64.2
HOTS (Lagorce et al., 2017)	21.0
DART (Ramesh et al., 2019)	66.4
YOLE (Cannici et al., 2019)	70.2
EST (Gehrig et al., 2019)	81.7
SSC (Graham et al., 2018)	76.1
Asynchronous sparse CNN (Messikommer et al., 2020)	74.5
Collapsed images with ANN	78.01

*Our method is the second best*.

Our method of just summing up spikes over time (therefore getting rid of the time representation) is able to obtain comparable to state of the art accuracy compared to neuromorphic datasets. Although few SNNs give marginal improvement over our method, it is important to note that we are not trying to beat other algorithms by building bigger ANN systems, and optimizing the algorithm. Our aim is to simply show that there is no significant reduction in accuracy using a method that does not use temporal information encoded in the timings of spikes at all. The implications of this result are further discussed in the section 7.

Finally we tested on DvsGesture which was not derived from static images but dynamic hand and arm movements. Without the *Other* class, the dataset has 10 classes. It was therefore trained on a CNN that was identical to the one used for training N-MNIST. We obtained an accuracy of 71.01% on the ANN which is far worse than the state of the art results obtained by other algorithms as seen in Table 5. Since DvsGesture has information coded in spike timings (as it is obtained by dynamic movements) it requires an SNN to make efficient use of this information encoded in spike timings to obtain better accuracy than ANNs.

**Table 5:** Comparison of DvsGesture performance with our method and other state of the art algorithms.

**Method**	**DvsGesture (%)**
Maro and Benosman (Maro and Benosman, 2019)	96.6
Yang et al. (Yang et al., 2019)	97.4
SLAYER (Shreshtha and Orchard, 2018b)	93.64
CNN on TrueNorth (Amir et al., 2017)	96.49
Collapsed images with ANN	71.01

The conclusion of this experiment is the following: While neuromorphic datasets derived from static images have excellent performance on par with state of the art on ANNs, neuromorphic datasets derived from actual movements perform far worse on ANNs than the state of the art accuracy obtained by SNNs.

## 4. Spiking Neural Network

The rest of the experiments in this paper are run on spiking neural networks (SNN) using spike timing dependent plasticity (STDP) learning rule. In this section, we will describe the SNN that is used for the experiments. The SNN algorithm in this paper closely follows (Diehl and Cook, 2015), but has been modified to suit the N-MNIST dataset. For a detailed description of these modifications, refer to Iyer and Basu (2017).

### 4.1. Network Architecture

The input layer contains 34 × 34 neurons (one neuron per image pixel in N-MNIST). Each input neuron projects to all neurons in the *excitatory layer* with weights *W*^*xe*^. The *excitatory layer* has *N*_*e*_ neurons which have a one-to-one connectivity with *N*_*i*_ neurons in the *inhibitory layer*. Note that *N*_*i*_ = *N*_*e*_. When a neuron spikes in the *excitatory layer* it will activate the corresponding neuron in the *inhibitory layer*. Each inhibitory neuron inhibits all neurons in the *excitatory layer* except the one that it has afferent excitatory connection with. The net effect is lateral inhibition.

**Figure 3:**
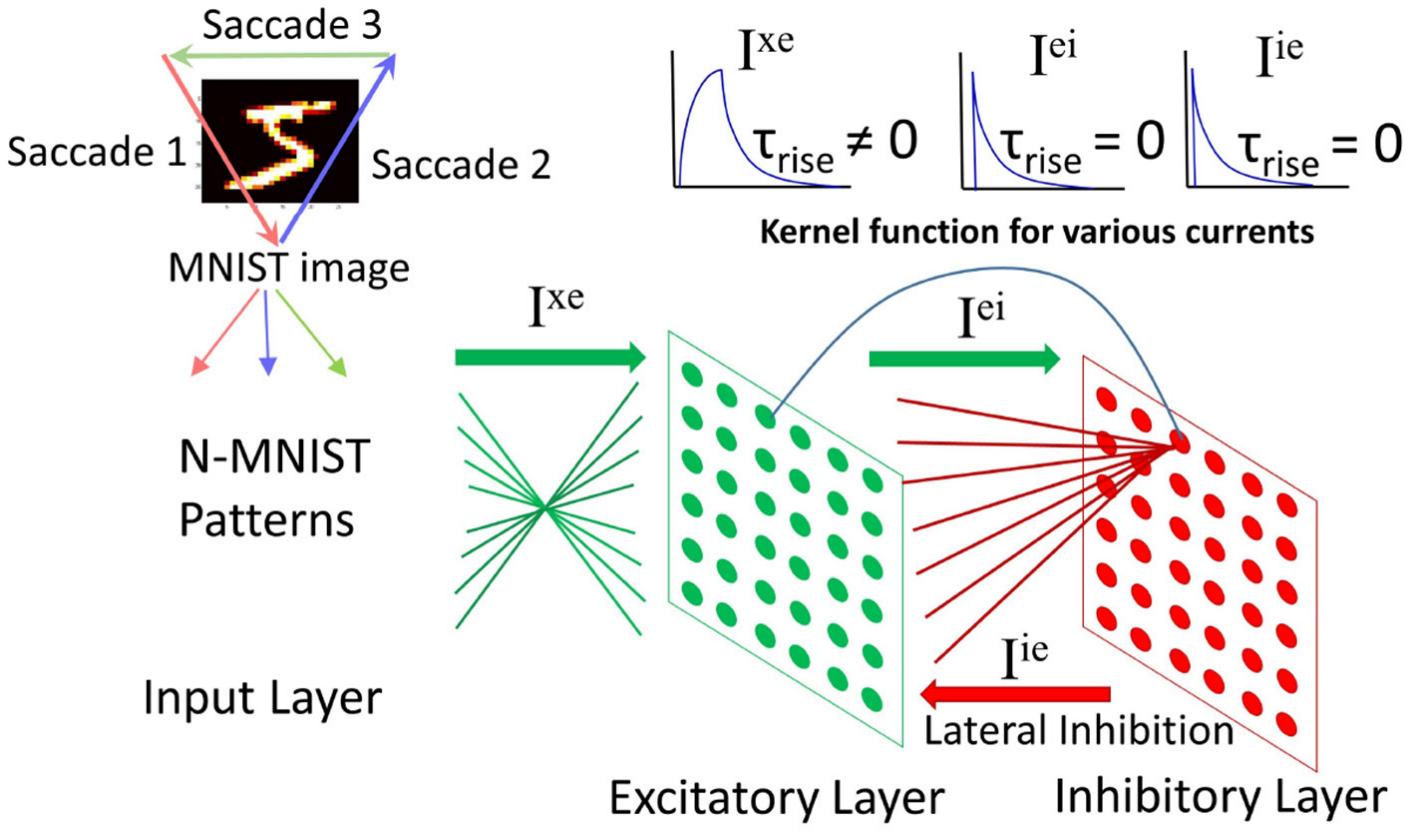
Spiking neural network architecture: (modified from Iyer and Basu, 2017, Figure 2). The N-MNIST patterns, each pattern representing a *saccade* project to the excitatory layer. There is an all-to-all connection from the input to excitatory layer. The excitatory layer has a one-to-one connection with the inhibitory layer. Upon firing, an excitatory neuron activates its corresponding inhibitory neuron, which in turn inhibits all excitatory neurons except the one it received excitatory connections from. Top right: Kernel functions for the currents in the network—*I*_*xe*_ has a gradual rise in current followed by a gradual fall. The other two currents, *I*_*ei*_ and *I*_*ie*_ have an instantaneous rise in current followed by a gradual fall.

The system architecture is shown in Figure 3. More information on the network dynamics can be found in Iyer and Basu (2017).

### 4.2. Learning

The learning function follows from Diehl and Cook (2015). When there is a postsynaptic spike, the synaptic weight update Δ*w* is:

(3) $$\begin{array}{r} \Delta w=\eta \left(x_{pre}-x_{tar}\right){\left(w_{max}-w\right)}^{\mu } \end{array}$$

where *x*_*pre*_ is the presynaptic trace, *x*_*tar*_ is the target value of the presynaptic trace at the moment of postsynaptic spike, *η* is the learning rate, *w*_*max*_ is the maximum weight, and *μ* determines the dependence on the previous weight. See Diehl and Cook (2015) for more details.

When a presynaptic spike arrives at the synapse, the presynaptic trace, *x*_*pre*_ is increased by Δ*x*_*pre*_, and decays exponentially with the time constant *τ*_*x*_*pre*__.

### 4.3. Threshold Adaptation

The threshold adaptation mechanism used here is identical to that employed by Diehl and Cook (2015). In order to prevent any single neuron in the excitatory layer from dominating the response pattern, it is desirable that all neurons have similar firing rates at the end of training. Therefore, the neuron's firing threshold *V*_*th*_ is adapted as follows:

(4) $$\begin{array}{r} V_{th}=v_{thresh}+\theta \end{array}$$

*V*_*th*_ has two components, a constant *v*_*thresh*_ and a variable component, *θ*. *θ* is increased by Δ*θ* every time a neuron fires, and decays exponentially with a very large time constant, $\tau_{\theta }=10^{7}ms$, rendering the decay negligible during the simulation. Therefore if a neuron spikes more, its threshold is higher, requiring more input for the neuron to spike.

### 4.4. Pattern Presentation

If for any pattern presentation there is no output spike, *A*^*xe*^, the EPSC of a single neuron is increased by Δ*A*^*xe*^ and the pattern is presented again. This is repeated till there is an output spike.

### 4.5. Neuron Label Assignment

Once the training is done, the training patterns are presented again to the learnt system. Each neuron is assigned to the class that it most strongly responds to. This neuron assignment is used in calculating the classification accuracy. Note that class labels are only used in this step, and not for training.

### 4.6. Parameters

The values of most parameters in this SNN follow (Diehl and Cook, 2015). These include *V*_*rest*_, *v*_*thresh*_ and *V*_*reset*_ in the *excitatory* and *inhibitory* layers. Since we present each pattern one after another, the presentation time for N-MNIST is 105 ms, equivalent to the time taken for one saccade in the N-MNIST dataset. For DvsGesture, we take only the first 1,450 ms of the pattern to classify the dataset, as has been done earlier (e.g., Stewart et al., 2020) and this is the presentation time. As presynaptic spike rates vary throughout pattern presentation, the output neuron must spike only at the end of the presentation (see Iyer and Basu, 2017 for more details). Therefore, *τ*_*M*_, the membrane time constant of each excitatory neuron is adjusted such that there is only one output spike (see Iyer and Basu, 2017 for additional information) occurring toward the end of pattern presentation. The value of *τ*_*STDP*_ used is more than double the presentation time for both datasets. After each pattern presentation, all values except *W*_*xe*_ and *θ*_*e*_ are reset, as is done in Diehl and Cook (2015). Diehl and Cook (2015) do this by having a period of inactivity for 150*ms* in between pattern presentations. However, it would be more biologically plausible to not reset these parameters, and this is something we would explore in our future work.

This system has been used with large values of *τ*—this approximates a rate based system that sums up the spikes. Hence we term the system *rate-dependent STDP*, or *RD-STDP*.

For the *Design Space Explorations* (see section 5) learning rate—*η* and amplitude of threshold adaptation—Δ*θ* are adjusted accordingly.

### 4.7. Temporal Spiking Neural Network

**Figure 4:**
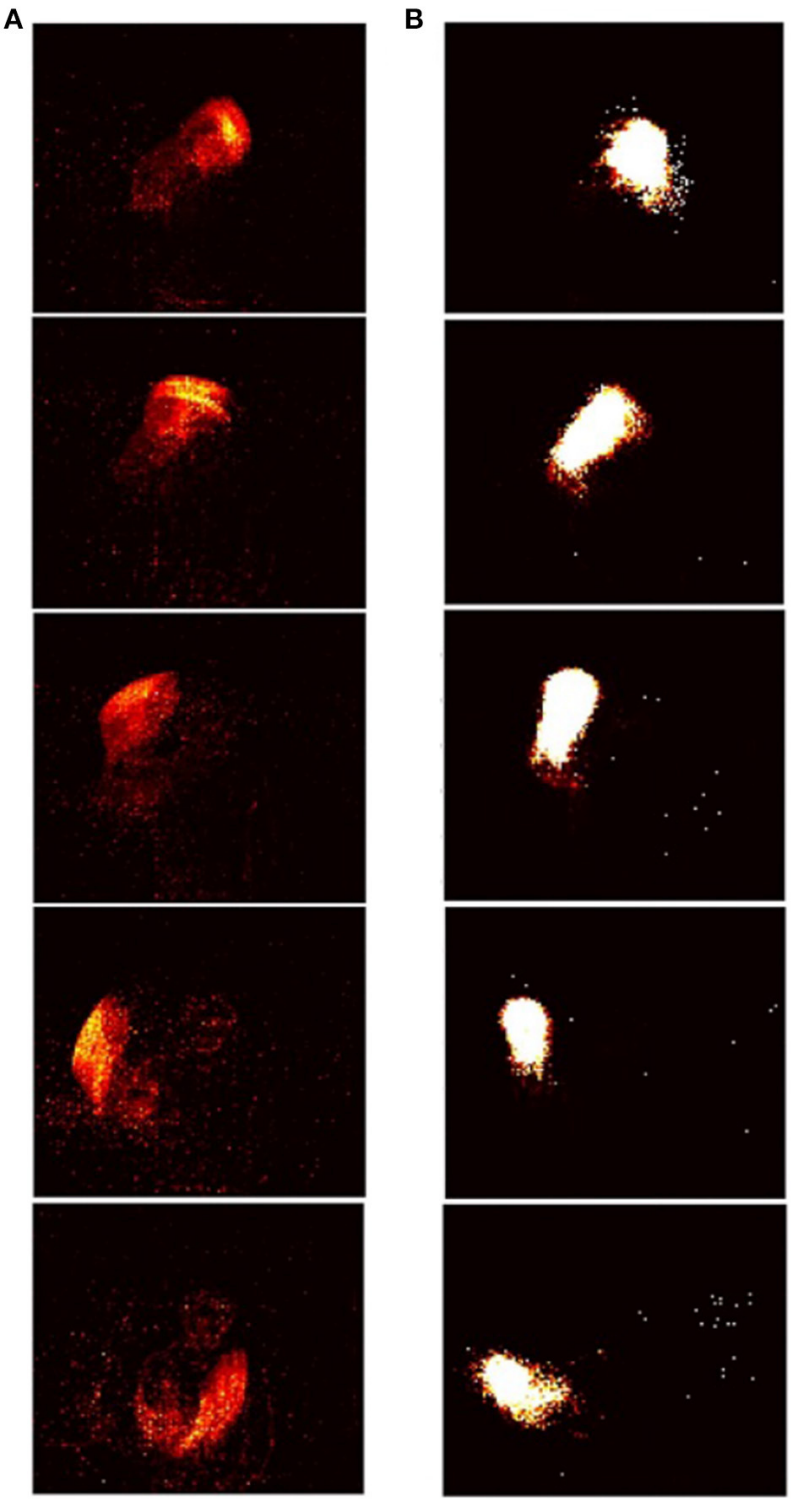
Input-output sequences in STDP-tempotron: (reproduced from Iyer and Chua, 2020, Figure 1). **(A)** Sequence of frames created from input spikes. Input spikes collapsed over every *m* ms, where *m* is $\frac{1}{10}th$ the presentation time. The images from top to bottom depict a pattern from the class *right hand clockwise*. **(B)** The images from top to bottom show the sequence of output neurons that spike in response to the input. Each output neuron is represented by weights from the input, and rearranged on a 128 × 128 grid as in Figure 5. As can be seen, the sequence of output neurons that fire on the right, look very similar to the input spikes on the left.

**Figure 5:**
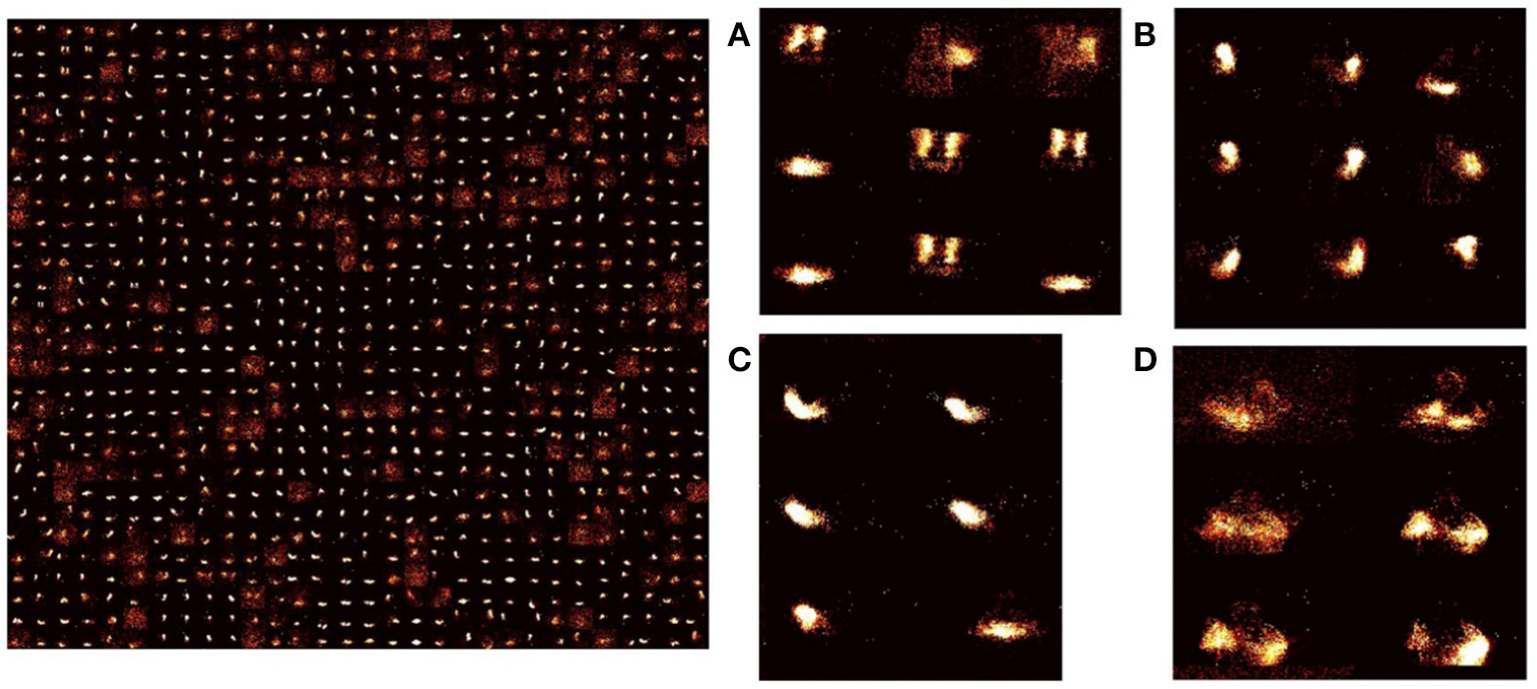
Learned weights in STDP-tempotron: (reproduced from Iyer and Chua, 2020, Figure 2) Input-excitatory weights of a 900 output neuron network after training—left: weights from input to each excitatory neuron is arranged as 128 × 128 matrix to visualize the input learnt. These individual neuron weights are arranged on a 30 × 30 grid. Right: **(A–D)** Zoomed snapshots of the image on the left. As can be seen the weights learn temporal snapshots of different actions. These snapshots can be used as raw material for producing actions from different classes. **(A)** Some images are a part of *clapping* and others, *arm rolling*, and others, *left hand wave* or *left hand clockwise or counterclockwise* movements, **(B)** Can be part of *left-hand waving* or *left hand clockwise or counterclockwise* movements, **(C)** can be a part of *right-hand waving* or *right hand clockwise* or *counter clockwise* movements, and **(D)** can be part of *arm rolling* or *air drums*. Similar actions are grouped together in space due to SOM functionality.

The RD-STDP network described above has been successful at classifying MNIST (Diehl and Cook, 2015) and N-MNIST (Iyer and Basu, 2017). However, for each pattern, only one output neuron spikes (either one or many spikes) and learns the pattern. For datasets where the pattern changes temporally during pattern presentation, and this additional temporal information encoded in spike timings is important in classifying the data, one output spike that learns an entire pattern is inadequate. A sequence of output spikes each of which learn subpatterns of the temporal pattern would be necessary (see Figures 4, 5). Patterns should be classified based on this entire sequence.

In Iyer and Chua (2020), we have modified the system described above, to classify temporal patterns. We add the Self-Organized Feature Map (SOM) functionality and the tempotron to the current network. The tempotron is a biologically plausible learning rule for classifying spatiotemporal patterns, and can classify a sequence of input spikes.

Given below is the summary of modifications we made to the RD-STDP to enable it to classify temporal data.

- *τ*_*M*_ and *τ*_*xpre*_ have been adjusted to be a fraction $\frac{1}{10}th$ of the pattern presentation time. After every *kms* where *k* is $\frac{1}{10}th$ the presentation time, all voltage traces, all currents and current traces, and synaptic traces are reset.
- In RD-STDP, when no spike occurs, *A*^*xe*^, (EPSC) is increased and the pattern is presented again (see section 4.4). However, here it is essential that the spikes occur in an online manner, as there are a sequence of spikes for each pattern. Therefore, *A*^*xe*^ is kept constant.
- The sequence of spikes produced by the STDP system are then classified by a tempotron (Gutig and Sompolinsky, 2006) in a supervised manner.

We hereby term the temporal version of the system *STDP* − *tempotron*. Note that the two systems are essentially the same. Some minimal features are added in order to classify temporal data. Also note that in STDP-tempotron, the clustering of neurons is completely unsupervised as in RD-STDP. Only the classification of output sequences occurs in a supervised manner.

As RD-STDP classifies information by integrating information with large *τ*_*STDP*_ time constants, it can only classify based on spatial information. On the other hand, with smaller *τ*_*STDP*_ values, and added capabilities to classify sequences, STDP-tempotron can classify based on spatio-temporal info.

In the sections that follow we describe the experiments that use the RD-STDP and STDP-tempotron described above.

## 5. Experiment: Design Space Exploration in SNN to Explore Temporal and Rate-Based STDP Regimes

**Figure 6:**
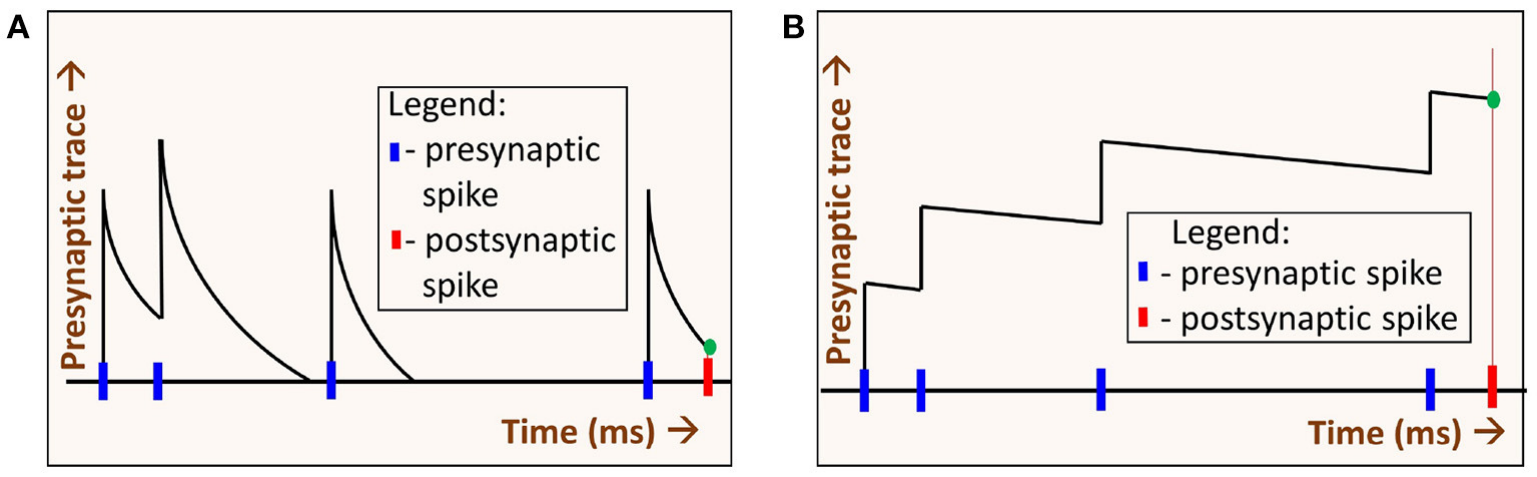
Two regimes of STDP operation: By varying the decay time constant of the presynaptic spike trace *τ*_*xpre*_ we get two regimes of STDP operation, **(A)** When *τ*_*xpre*_ is low, the presynaptic trace (*x*_*pre*_) decays quickly. The value *x*_*pre*_ at the time of the postsynaptic spike (green dot) depends on the time difference between the pre- and post-synaptic spikes. In this regime, spikes that occurred much earlier than the postsynaptic spike time have no impact on learning. **(B)** When *τ*_*xpre*_ is high, *x*_*pre*_ decays slowly. At the time of postsynaptic spike, *x*_*pre*_ (green dot) depends on the number of spikes (i.e., spike-count rate) alone. Precise presynaptic spike times do not have much impact on learning.

Spike-timing dependent plasticity (STDP) is a learning rule commonly used in SNNs for unsupervised learning. For the SNN experiments, we choose spike-timing dependent plasticity (STDP) for the following reason. Generally in STDP, weight updates are based on the precise difference between pre and postsynaptic spike times. When the synaptic trace time constants are increased, STDP operates in a regime whereby weight changes can be approximated by sum of pre-synaptic and post-synaptic spikes. One can intuitively understand this by assuming delta synaptic trace on one extreme, and perfectly integrated synaptic trace on the other extreme. The former would be highly sensitive to spike timing (they must occur at same time for weight changes), while the later would have weight changes proportional to spike counts of the neurons. These different modes due to presynaptic time constant (*τ*_*xpre*_) are illustrated in Figure 6. Hence STDP learning rule is highly suitable for our exploration, as it can operate in spike-time based as well as rate-based modes.

If N-MNIST has better performance in the rate-based regime, then precise spike timing in N-MNIST dataset would seem unnecessary for classifying it, to the extent that the experiment findings can be generalized. We think they can be for the reasons stated below.

It is hard to compare performance of an SNN trained using backpropagation against one trained using STDP, given the difference in network topology and learning algorithms. However, there is one commonality across both, and that is the use of synaptic trace in their learning rules (Gutig and Sompolinsky, 2006; Lee et al., 2016; Zenke and Ganguli, 2018). By tuning the time constants of the synaptic trace, one effectively tune the sensitivity of the SNN toward spike-timing. Hence, from this aspect, our results can be further generalized to all other SNN based learning algorithms whose weight update contains the synaptic trace.

In our experiments, we use STDP-tempotron that has a short synaptic trace time constant, along with added capabilities to classify sequences. RD-STDP on the other hand, has a synaptic trace that is larger than the presentation time which therefore approximates rate-based learning. Further, it has added capabilities to classify sequences.

In this experiment, we compare two datasets, N-MNIST with DvsGesture, as in section 3. We compare their performance on two systems, RD-STDP and STDP-tempotron. A dataset with no additional encoded in the timing of spikes is expected to perform better in RD-STDP, which can adequately classify the static spatial dataset. On the other hand, when a dataset has additional information contained in the timing of spikes, STDP-tempotron with its added capability is better than RD-STDP at classifying the dataset. So the hypothesis for our experiment is as follows.

*Hypothesis:* N-MNIST is expected to perform better on RD-STDP than on STDP-tempotron. However, DvsGesture is expected to perform better on STDP-tempotron compared to RD-STDP.

### 5.1. Methodology

The combination of the two algorithms and two methods lead to four experimental cases, (1) N-MNIST on RD-STDP, (2) DvsGesture on RD-STDP, (3) N-MNIST on STDP-tempotron, and (4) DvsGesture on STDP-tempotron.

In order to compare different algorithms and datasets, trials have been performed on a range of parameter values for important parameters. These parameters are:

1. *η*, Learning rate—Higher values of *τ*_*xpre*_ would result in higher values of the presynaptic trace, *x*_*pre*_ as individual spike traces would decay slowly. This results in an accumulation of individual spike traces over time. This, in turn, would lead to higher weight updates [see the learning rule (Equation 3) in section 4.2]. To ensure that results are not biased due to more learning in the system, we vary *η*.
2. Δ*θ*, Amplitude of threshold adaptation (see section 4.3)—Threshold adaptation is done to prevent some neurons from dominating the learning and distributing the receptive field of input patterns over all neurons. However, if threshold adaptation occurs very slowly compared to the learning rate, this purpose will not be served. If, on the other hand, the threshold of a neuron is increased very quickly before it even learns, then during training, no useful learning will take place. We therefore change Δ*θ* along with *η*.
3. *A*_*xe*_, EPSC (only for STDP-tempotron)—Although in RD-STDP, the EPSC increases if there is no output spike (see section 4.4), in STDP-tempotron, it is kept constant (see section 4.7). For some values of *A*_*xe*_, there is no output spike, while others will have more spikes. Since this value affects the accuracy, this is one of the parameters of consideration for STDP-tempotron.
4. *N*_*e*_, the number of output neurons in the network, henceforth called *Network Size*. Since we are using datasets of different sizes (DVSGestures has only 732 training patterns, while N-MNIST has 60,000 training patterns), and a one layer network, different network sizes would be optimal for different experimental cases.

We perform two design space explorations—*DSE*1 for the first three parameters, and *DSE*2 for the fourth parameter. For the first two parameters, we do not know the optimal values. Hence, we do a systematic design space exploration of *all possible combinations* of the first three parameters for STDP-tempotron and RD-STDP. The first two parameters are varied on a *logarithmic* scale. For the third parameter, we first work logarithmically to find a ball park current that yields enough spikes for classification, and then vary this current value on a linear scale. This is because having very small current values will not yield enough spikes for classification. We perform the *DSE*1 experiment to find the best set of values for the first three parameters.

For the fourth parameter, i.e., network size, we can theoretically determine a network size that can be used without loss of generality, for optimum results. This is given as follows, and summarized in Table 6.

- DvsGesture SNN—49 neurons—There are very few patterns in the DvsGesture dataset. Since the large membrane time constant and large STDP time constant collapse the image and a single spike is learnt for each pattern, there will not be many weight changes within the pattern. Due to this, the network size needs to be low so that parameters are adequately trained.
- DvsGesture STDP-tempotron 900 neurons—although the number of patterns are low, there are many spikes, and so the weights essentially learn subpatterns within a pattern. Therefore, a larger size is deemed more suitable in order to classify all the subpatterns.
- N-MNIST RD-STDP—400 neurons—The dataset size is very large and therefore, we do not need to use small networks.
- N-MNIST STDP-tempotron—400 neurons—The subpatterns are not expected to be very different from the main pattern. Therefore, we do not see the need for larger networks either.

**Table 6:** Network size used in each of the experimental cases.

**Dataset**	**Algorithm**	**Network size**
DvsGesture	RD-STDP	49
DvsGesture	STDP-tempotron	900
N-MNIST	RD-STDP	400
N-MNIST	STDP-tempotron	400

*DSE*1 is performed using the network sizes described above. An obvious concern is that network sizes chosen above might bias the results. To obviate this concern, we perform another experiment, *DSE*2. For *DSE*2 we hypothesize that having different network sizes from those described in the previous list will not change the results.

We therefore, perform 2 design space exploration experiments, *DSE*1 and *DSE*2. The purpose of *DSE*1 is to do a systematic hyperparameter search to find the set of the first three parameters that yield the best results for each of the four cases, using theoretical values of the fourth parameter. The purpose of *DSE*2 is to ensure that there is no network size that is better than the theoretical values we have specified in *DSE*1. We are able to perform *DSE*2 separately as each of the other three parameters can be adjusted for network size as follows—(1) *A*_*xe*_ is independent of network size as it acts on each individual neuron. (2) The learning rate *η* and amplitude of threshold adaptation, Δ*θ* can be adjusted for network size, which we examine in Iyer and Chua (2020), section 3A.

The reason why we perform *DSE*2 as a separate experiment instead of including network size as an additional parameter in experiment 1 is largely to reduce computational costs. We use the theoretical values of the fourth parameter to perform a design space exploration of a much smaller parameter space (having only three parameters), and then perform the much smaller *DSE*2 experiment to ensure that the usage of theoretical values of the fourth parameter in *DSE*1 does not bias the results.

We describe the two experiments as follows.

*DSE1*:

Hypothesis: N-MNIST is expected to perform better on RD-STDP than on STDP-tempotron. However, DVSGestures is expected to perform better on STDP-tempotron compared to RD-STDP.

By doing a hyperparameter search of the parameters, we will be able to compare the performance on the four systems and verify the above hypothesis. Here we focus on all parameters, other than *Network Size*, which would be dealt with in *DSE*2. A summary of the parameters, the corresponding algorithm(s) and experiment wherein they are studied is given in Table 7.

**Table 7:** Summary of parameters used in *DSE*1 and *DSE*2.

**Parameter**	**SNN algorithm**	**Scale of variation**	**Experiment**
Learning rate, *η*	RD-STDP & STDP-tempotron	Logarithmic	DSE1
Threshold adaptation amp., Δ*θ*	RD-STDP & STDP-tempotron	Logarithmic	DSE1
EPSC current, *A*_*xe*_	STDP-tempotron	Find ballpark and vary linearly	DSE1
Network size, *N*_*e*_	RD-STDP & STDP-tempotron	Linear	DSE2

Learning rate, *η* and spike frequency adaptation rate Δ*θ* are varied on a logarithmic scale to ensure that we cover all possible ranges of activity. The values of *η* used are {0.0005, 0.005, 0.05, 0.5}. The values of Δ*θ* used are {1, 0.1, 0.01}*mV*. For STDP-tempotron, an additional parameter was explored, and that is *A*_*xe*_. We choose the parameters for *A*_*xe*_ as follows. It was noted that for DvsGesture, an *A*_*xe*_ value of 0.5*nA* is enough to get at least one output spike for most patterns, and classify the dataset. However, for N-MNIST, the *A*_*xe*_ value had to be around 5*nA* before there was at least one output spike for most patterns. For *A*_*xe*_ values less than this, many patterns did not get any output spikes, and the dataset could not be classified. For *A*_*xe*_ values are varied on a linear scale, around the preliminary values stated earlier,—for DvsGesture, *A*_*xe*_ values used are {0.1, 0.3, 0.5, 0.7} nA, but for N-MNIST, the *A*_*xe*_ values used are {1, 3, 5, 7} nA.

The best results for each of the four experimental cases are given in Table 8.

**Table 8:** *DSE1 Results:* The best results of DSE1 for the two datasets, DvsGesture and N-MNIST on the two algorithms, RD-STDP and STDP-tempotron.

	**RD-STDP (%)**	**STDP-tempotron (%)**
DvsGesture	53.18	**59.11**
N-MNIST	**83.89**	76.13

*Bold values indicates the better accuracy for each algorithm*.

As can be seen, RD-STDP performs better than STDP-tempotron at N-MNIST, while STDP-tempotron performs better than RD-STDP at DvsGesture. Therefore, the hypothesis for DSE1 has been satisfied.

*DSE2:* This section describes the protocol for DSE2.

Our hypothesis for DSE2 is:

1. DVSGestures—no RD-STDP of any network size can exceed the best performance of STDP-tempotron obtained in DSE1.
2. N-MNIST—no STDP-tempotron of any network size can exceed the best performance of RD-STDP obtained in DSE1.

- To verify DSE2 Hypothesis 1, we take the best performing RD-STDP in DSE1, adjust for network size, and test DVSGestures on RD-STDP networks of different sizes.
- To verify DSE2 Hypothesis 2, we test the best performing STDP-tempotron in DSE1, adjust for network size, and test N-MNIST on STDP-tempotron networks of different sizes.

The protocol for DSE2 experiments is as follows.

(a) Following from Hypothesis 1 for DSE, we take the parameters for the best performing RD-STDP in DVSGestures. (b) Following from Hypothesis 2 for DSE, we take the parameters for the best performing STDP-tempotron in N-MNIST.
We modify Δ*θ* and *η* for a modified network size according to the protocol we describe in Iyer and Chua (2020), section 3A.
We choose network sizes that are evenly distributed from 49 to 900. The network sizes are chosen to be relatively even squared numbers between 49 and 900, and are—{49, 225, 400, 576, 729, 900}
We ran the system and noted the accuracies. These are given in the table below.

In *DSE*2, after running on networks with different sizes, we see in Table 9 that the best performing STDP-tempotron on N-MNIST has an accuracy of 77.43%. However, N-MNIST on RD-STDP has a best performing accuracy of 83.89%. Thus, Hypothesis 1 of *DSE*2 is satisfied. Also, the best performing RD-STDP on DvsGesture has an accuracy of 53.18%, and is for the smallest network size of 49, as seen in Table 10. This accuracy is less the best performing STDP-tempotron for DvsGesture which has an accuracy of 59.11%. Therefore, Hypothesis 2 of *DSE*2 is satisfied. Overall, *DSE*1 and *DSE*2 show that indeed, STDP-tempotron, a temporal algorithm with short synaptic trace time constants works better with DvsGesture. However, N-MNIST performs better on RD-STDP algorithm. Also we note that in N-MNIST larger networks have better accuracy, while in DvsGesture this is not necessarily the case, as we mentioned earlier.

**Table 9:** DSE2 results on N-MNIST using STDP-tempotron networks of different sizes.

**No. of output neurons**	**Accuracy (%)**
49	60.14
225	73.19
400	76.13^*^
576	76.67
729	**77.43**
900	77.19

*The blue asterisk indicates (not indicate) and the bold indicates the best results*.

**Table 10:** DSE2 results on DvsGesture using RD-STDP networks of different sizes.

**No. of output neurons**	**Accuracy (%)**
49	53.18^*^
225	19.71
400	12.94
576	17.66
729	25.46
900	24.84

*The blue asterisk indicates (not indicate) and the bold indicates the best results*.

It is evident from these experiments that DvsGesture performs better with an algorithm that is suitable for temporal datasets, and has smaller synaptic traces. The absolute results in DvsGesture dataset are not very good due to overfitting (as discussed further in Iyer and Chua, 2020), but the trends clearly show that the rate based system performs more poorly in classifying the dataset. On the other hand, N-MNIST shows the opposite trend and better results are obtained on an STDP system that approximates rate based calculations. Indeed, we see in the next section that the unsupervised results on N-MNIST by this system is indeed state-of-the-art. This indicates that there is no additional information in the time domain in the N-MNIST dataset necessary to classify it.

### 5.2. Further Results With N-MNIST on STDP

We performed further experiments with N-MNIST on STDP to improve the results.

As results are on the *logarithmic* scale, there may be intermediate parameter values that give better results. To check this, we repeat the experiments with just one epoch for parameters around the vicinity of the best results. We determine that the best results are given by values Δ*θ* = 0.2*mV*, *η* = 0.05, *τ*_*xpre*_ = 215*ms*). Using these parameters, we repeated this experiment for 3 epochs. Finally, we repeated this procedure for training 6 separate 400 neuron networks—each of the 3 saccades with both ON and OFF polarities. For each test pattern, all 6 networks gave a class prediction and we took a majority vote. The results obtained are summarized in Table 11.

**Table 11:** Further results on N-MNIST with the best parameters in linear scale.

**Specifications**	**Accuracy (%)**
1 epoch, 1 saccade, ON polarity	82.46
3 epochs, 1 saccade, ON polarity	89.87
3 epochs, 6 separate networks, 3 saccades, ON and OFF polarities, 2,400 output neurons	91.78
**Results with Diehl and Cook (****2015****)**	
400 output neurons	87.0
1600 output neurons	91.9

*This is compared with MNIST results obtained in Diehl and Cook (2015)*.

The results obtained by the rate based STDP on N-MNIST are highly comparable similar STDP based methods. We compare the system with a similar STDP system on MNIST (Diehl and Cook, 2015—see Table 11). It is not surprising to see a slight deterioration in N-MNIST over MNIST due to noisy and more realistic input.

We are performing the rest of the STDP experiments with just one epoch, and comparing to the results of this experiment carried out with one epoch.

## 6. Experiment: STDP With Fixed Postsynaptic Spike

Earlier we noted that rate based STDP regime yields the best accuracy results indicating that presynaptic spike times do not affect the accuracy. If learning is dependent on purely spike rates alone, we postulate that the precise timing of postsynaptic spikes should not affect the accuracy either. So if we fix the postsynaptic spike to occur at a certain time for every pattern, there should not be a fall in accuracy.

**Figure 7:**
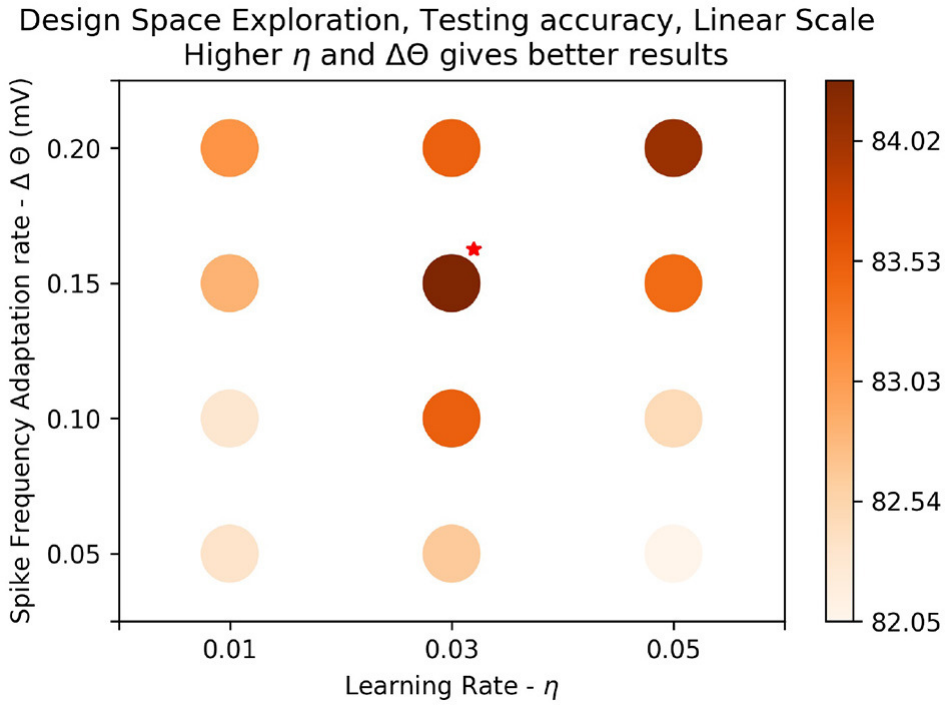
Results on *linear scale*: The best test accuracy in the *log scale*, (*τ*_*xpre*_ = 215*ms*, *η* = 0.05, Δ*θ* = 0.1*mV*) was taken. Values of the parameters, Δ*θ* and *η* around the best result were plotted on linear scale to determine if there were better results around the parameter space. Training was done over one epoch. We see that generally results get better as learning rate (*η*) gets higher and amplitude of threshold adaptation (Δ*θ*) gets higher. The best results are marked with a red star on the top right. We get a slight improvement in accuracy, 84.51%.

In this experiment we train the system using the parameters for the best results seen in the previous experiment (Figure 7: Δ*θ* = 0.2*mV*, *η* = 0.05, *τ*_*xpre*_ = 215*ms*), and record the postsynaptic spike time for each pattern. We then find the average of the postsynaptic spike time over all patterns, t^*^.

We re-start and repeat the training fixing the postsynaptic time to be t^*^. As we have a Winner-Take-All network, and the neuron that is the first to spike wins, we do not enforce the neuron to spike at time t^*^, but the network learns *as if* the spike occurs at t^*^. Therefore, we take the presynaptic spike traces at time t^*^ to calculate the weight updates.

The accuracy for this experiment is 84.10% after one epoch. This is even better than the best accuracy results in the DSE experiment 5 which is 82.46%. The high accuracy indicates that performance is not dependent on the precise timing of the postsynaptic spike either.

## 7. Experiment: Spike Rate Dependent STDP

In the previous experiments, we examined the performance of a simple ANN and the SNN (both rate based and time based) on the N-MNIST dataset. Both the ANN and the rate based SNN use time-averaged firing rates (Figure 1) for classification. In this final experiment we examine the effect of instantaneous population rate (Figure 1) on performance.

We note that events recorded by the ATIS sensor are relatively sparse at the beginning and end of a saccade. Most events occur in the middle of a saccade. So, we hypothesize that the middle of the saccade is the time period where the information is the most abundant. Events that happened at the beginning and end of the saccade could be regarded as noise. From this, we hypothesize that by (1) using an *engineered* STDP function—i.e., an STDP function that is based on the peristimulus time histogram (PSTH) of the training data, and (2) fixing the postsynaptic spike time at the end of the pattern presentation, we will not experience a decrease in performance. Such an STDP function is completely independent of the pre and postsynaptic spike time differences, and is governed by the instantaneous population spike rates alone. If the above hypothesis is correct, then spike times of individual neurons are unnecessary. Instantaneous population spike rates adequately characterize the dataset.

The STDP function is created as follows. Each pattern *p* can be represented as a set of spike trains, with one spike train for each pixel. The spike train for pattern *p* and pixel *x* is represented as $s^{x,p}=\left\{t_{1}^{x,p},t_{2}^{x,p},...t_{n}^{x,p}\right\}$, where each of the elements represents the time at which the corresponding event occurred. Note that *t*_1_, …, *t*_*n*_ are in the range [0, 105] ms, (first saccade) and are all *ON* events.

The total number of events that occurred over all patterns *p* at all pixels *x* at the instantaneous time between *t* and Δ*t* is:

(5) $$\begin{array}{r} H^{\prime }\left(t\right)=\underset{p}{\sum }\underset{x}{\sum }a_{i}^{x,p} \end{array}$$

(6) $$a_{i}^{x,p}=\{\begin{array}{ll} 1, & t\leq t_{i}^{x,p}\leq \Delta t,t_{i}^{x,p}\in s^{x,p};\\ 0, & \text{otherwise}. \end{array}$$

(7) $$\begin{array}{r} H\left(t\right)=\frac{H^{\prime }\left(t\right)}{N_{patterns}} \end{array}$$

*H*(*t*) is then scaled and biased as follows:

(8) $$\begin{array}{r} h\left(t\right)=aH\left(t\right)+b \end{array}$$

Parameters *a* and *b* are chosen so that the STDP function *h*(*t*) fulfills the following conditions:

- The area of LTD is greater than the area of LTP—this is to ensure network stability (Song et al., 2000).
- The weight updates are of similar magnitude to that of the learning rule described in section 4.2. This is determined empirically using the first few patterns so as to determine *a* and *b*, so that learning rate would not be the varying factor in the classification accuracy obtained in experiments.

**Figure 8:**
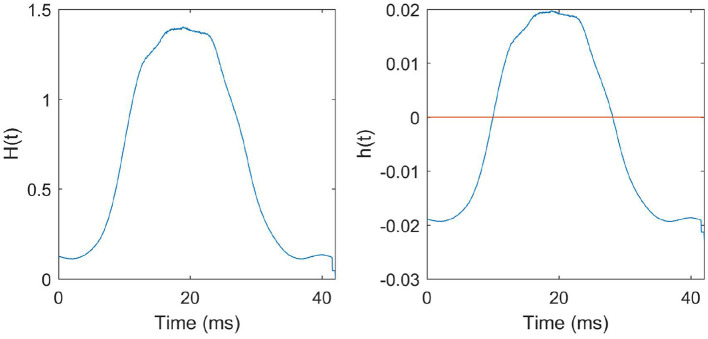
STDP function derived from the training data: **Left:** *H*(*t*) is the average number of presynaptic neurons spiking at instantaneous time *t* in a pattern. The function *H*(*t*) derived from all the training patterns is given in the figure. **Right:** *h*(*t*) is the STDP curve obtained after scaling and biasing *H*(*t*) to ensure stability and to preserve the learning dynamics described in the previous sections.

The function *H*(*t*) that we derived from the N-MNIST training data and the corresponding STDP function *h*(*t*) that we obtained are given in Figure 8.

From the STDP function *h*(*t*) we calculate the presynaptic trace *x*_*pre*_ for a pattern *p* as follows:

(9) $$x_{pre}^{x,p}=\{\begin{array}{ll} \sum_{i=1}^{i=n}h(t_{i}^{x,p}), & n>0\\ -x_{tar}, & \text{otherwise}. \end{array}$$

So when a neuron fires one or more spikes, the resultant value *x*_*pre*_ is the sum of the values of *h*(*t*) for all time instances *t* where spikes occurred. Equation (9) also has a depression component. When a neuron does not spike at all, there is a LTD of −*x*_*tar*_. This is similar to section 3, Equation (3), where, in the absence of spikes, a neuron gets depressed by the same amount −*x*_*tar*_. The LTD component is introduced in Equation (9) to keep the learning dynamics similar to that of section 4.2. This addition of depression does not negate the purpose of this experiment—the STDP curve is still dependent on the presynaptic spike rate.

The learning rule is similar to that of Equation (3) in section 4.2:

(10) $$\begin{array}{r} \Delta w=\eta x_{pre}{\left(w_{max}-w\right)}^{\mu } \end{array}$$

where *x*_*pre*_ is the presynaptic trace, *η* is the learning rate, *w*_*max*_ is the maximum weight, and μ determines the dependence on the previous weight.

We trained the SNN using this learning rule above for one epoch, and we obtained an accuracy of 85.45%. Good performance was obtained on an PSTH derived STDP function. Postsynaptic spike time was also fixed. This indicates that precise time differences between pre and postsynaptic times are not necessary to classify the N-MNIST dataset.

## 8. Discussion

Given in Table 12 is a summary of the experiments conducted and their conclusion.

**Table 12:** This table shows the conclusions of the experiments conducted in this paper.

**No**.	**Algorithm**	**Datasets**	**Experiment**	**Conclusion**
1.	ANN–CNN described in Table 2	N-MNIST, N-Caltech101, DvsGesture	Spikes are summed over the presentation duration and collapsed into images. Then they are trained using an ANN.	The ANN obtains comparable to state-of-the-art results on N-MNIST (99.23%) and N-Caltech101 (78.01%). However, ANN performs significantly worse than state-of-the-art on DvsGesture (71.01%), as it cannot handle the spatiotemporal information. Hence, we conclude that N-MNIST and N-Caltech101 does not have additional information contained in the timing of spikes necessary to classify the dataset.
2.	RD-STDP and STDP-tempotron	N-MNIST and DvsGesture	Comparison of rate-based and temporal STDP algorithms on the two datasets	While the rate-dependent RD-STDP obtains very good performance on N-MNIST (83.89%), it is unable to do as well in DvsGesture (76.13%). In contrast, STDP-tempotron performs better in DvsGesture (59.11%), but worse for N-MNIST (53.18%). We conclude that while DvsGesture has spatio-temporal information, and therefore needs STDP-tempotron, N-MNIST does not have additional information in the time domain necessary to classify it.
3.	RD-STDP	N-MNIST	Fixing the output spike time	Despite fixing the output spike time, the system performs well (84.10%), demonstrating that precise timing of spikes are not useful at all in N-MNIST
4.	Populate rate dependent plasticity (new rule)	N-MNIST	A new STDP curve was devised based on summing up spikes over the population—the instantaneous population firing rate (The second definition of firing rate—see section 1, Paragraph 2)	Despite using an STDP curve based on the population spike rates alone, the system is able to give good performance on N-MNIST (85.45%). This demonstrates that spike timing is not important in classifying N-MNIST.

In this paper, we wanted to evaluate if neuromorphic datasets obtained from Computer Vision datasets with static images are discriminative in the time domain. We started the study with both N-MNIST and N-Caltech101, and performed several more experiments on N-MNIST alone to evaluate the same. In section 3, we demonstrate that a simple 9-layer CNN achieves 99.23% accuracy on collapsed N-MNIST which is comparable to the best results obtained with SNNs. Using the same method described in section 3, we examine N-Caltech101 images in another paper by our group (Gopalakrishnan et al., 2018). Here, we use a pre-trained VGG-16 (on Imagenet datasets), and retrain it using collapsed N-Caltech101 images (Gopalakrishnan et al., 2018, Figures 1C,D) and obtained the second best results on the dataset. In contrast in DvsGesture a neuromorphic dataset not derived from static images, our ANN has an accuracy of 71.01% which is far less than the 96.49% accuracy obtained by SNN, showing that SNN is preferred over ANN in datasets where additional temporal information contained in the timing of spikes is present. Results of this experiment, and comparison with other state-of-the-art algorithms are given in Tables 3–5. This in turn indicates that while collapsing the patterns in time does not affect the performance in N-MNIST and N-Caltech101, a similar trend is not obtained with DvsGesture dataset, which does significantly worse than state-of-the-art in the ANN. We noted that while DvsGesture performs better on the STDP-tempotron, the SNN network with short time traces and additional capability to classify a temporal dataset (59.11% on STDP-tempotron vs. 53.18% on RD-STDP), N-MNIST has better results on an RD-STDP, the SNN algorithm that has high synaptic trace time constants that approximate a summation of spikes (83.89% on RD-STDP vs. 76.13% on STDP-tempotron). Therefore, the rate-coded STDP system is adequate to classify and get very good results on N-MNIST.

We further showed that fixing the postsynaptic spike time gets a an accuracy of 84.10%, and the performance is not affected. Finally, we experimented on an instantaneous population rate based STDP function, and this achieved a performance of 85.45%. This shows that the instantaneous rate over a population of neurons fully characterizes the N-MNIST dataset. Collectively these experiments show that in the N-MNIST dataset, the precise timings of individual spikes are not critical for classification.

A central theme of our paper is the additional temporal information in precise spike timing and spike time differences. Therefore, it is necessary to highlight the importance of spike time coding. We gave some evidence on its importance in the introduction, and we begin this section with more biological evidence of spike time coding. Thorpe et al. (2001) has examined both time averaged rate and instantaneous population rate coding using Poisson spikes, the most prevalent rate coding scheme. Through simple statistical analysis he demonstrates that Poisson coding is not efficient enough to transmit detailed information about the level of excitation in a sensory receptor—and there are several studies detailing the importance of precise spike times in sensory systems: (1) Johansson and Birznieks (2004) points out that precise timing of the first spikes in tactile afferents encodes touch signals. Tactile perception is shaped by millisecond precise spike timing (Mackevicius et al., 2012; Saal et al., 2015). (2) In cats and toads, retinal ganglion cells encode information about light stimuli by firing only 2–3 spikes in 100 ms (Gabbiani and Midtgaard, 2001). (3) Studies have also shown the importance of spike timing in the vestibular system (Sadeghi et al., 2007) and somatosensory cortex (Harvey et al., 2013; Zuo et al., 2015). Finally, results in neuroprosthetics show that precise relative timing of spikes is important in generating smooth movement (Popovic and Sinkjaer, 2000). These studies suggest that when high speed of a neural system is required, timing of individual spikes is important. With the importance of precise spike timings, there are several neural coding theories that take spike timing into account—examples are time to first spike (Johansson and Birznieks, 2004; Saal et al., 2009), rank order coding (Thorpe et al., 2001; VanRullen and Thorpe, 2001; Kheradpisheh et al., 2018), polychronization (Izhikevich, 2006), coding by synchrony (Grey and Singer, 1989; Singer, 1999; von der Malsburg, 1999), predictive spike coding (Deneve, 2008) hypotheses.

As can be seen above, and in the introduction, there is a lot of evidence that spiking neurons use precise spike timing for effective coding and computation. In order to assess this ability in an SNN, a dataset is required to have additional temporal information in spike timings required for classification. In this paper, our hypothesis is that any neuromorphic dataset derived from static images, either by moving a camera or moving the images, does not contain relevant additional temporal information contained in the timing of spikes. We support this thesis through empirical means, by showing that systems using summation of spikes perform better than those that utilize the precise timing of spikes. The paper is divided broadly into two parts, first experiments with ANNs and second experiments with SNNs and STDP. Both parts of the paper are integral in supporting this hypothesis. The first part does so by showing that an ANN has comparable results to the state of the art SNNs when trained on collapsed neuromorphic dataset on N-MNIST and N-Caltech101, but the opposite trend is observed in DvsGesture, which performs significantly worse than state-of-the-art.

The second part explores why training with ANN obtains such good accuracy through STDP experiments in a SNN model. RD-STDP learns to integrate the spikes over large time windows (*τ*_*STDP*_), and uses these spike counts for classification. On the other hand, STDP-tempotron uses smaller *τ*_*STDP*_ and classify by looking for discriminatory spike patterns within a small time window. Results of this experiment are given in Tables 8–10. Currently, the network is shallow, with just one layer, and as a result, the performance of the current STDP-tempotron is limited. However, with a deeper network, in addition to discerning additional features, the tempotron can potentially learn a longer sequence, by integrating outputs of several discriminatory time windows. From the design-space exploration done, we drew insight and based on this new insight designed further experiments to prove that no additional temporal information in spike timings is required for good classification accuracy. We also reasoned why our approach is generalizable to SNNs in general. While comparing RNNs and SNNs, He et al. (2020) have also compared N-MNIST and DvsGesture, and also concluded that smaller time windows result in better performance for DvsGesture, but not N-MNIST.

From the insight drawn from above, we further show that when considering population rate coding (see section 1, paragraph 2, also Figure 1), there is a very regular pattern to the population spike rates. We derive a fixed learning curve based on the population rate code and is able to achieve good accuracy on the dataset. We would like to note that this learning filter is applied at the post-synaptic neuron after the input spike train has been presented. Hence the spike-timing of all neurons are disregarded and simply collapsed into a population rate code.

Ours is also the first unsupervised STDP SNN to be trained on image-derived neuromorphic dataset (i.e., RD-STDP), as has been described earlier (Iyer and Basu, 2017). We have produced a variant of this architecture suitable for classifying temporal data (STDP-tempotron—Iyer and Chua, 2020). Note that the tempotron is supervised. We compare these two different architectures, performing a systematic study with design space exploration. We show that while DvsGesture performs better with STDP-tempotron, N-MNIST is able to get very good results on the rate-coded RD-STDP system.

The second part of our paper hence shows that given spatio-temporal information encoded in the spike timing of a population of neurons, we can either sum up the spikes in the time domain or over the population, and both rate codes perform better compared to a STDP learning rule sensitive to precise spike timing. Hence both parts worked in tandem in support of the main contribution of our paper: part one to first pose the question (is additional temporal information contained in spike timings required for good classification accuracies for such neuromorphic datasets), and part two to show empirically that in fact better accuracies are obtained in N-MNIST but not DvsGesture when the spikes are summed up, hence answering the question posed.

As we have mentioned earlier, with a completely unsupervised STDP SNN, and with our temporal variant, where sequences are learnt in a supervised manner, we are not aiming to achieve state-of-the-art accuracy compared to other supervised learning methods; rather the tunable sensitivity to spike timing of STDP makes it useful for our study. Having said that, in the RD-STDP, we do achieve reasonable accuracies on N-MNIST compared to similar STDP based methods—with a 400 neuron network, we achieve 89.87% accuracy, while a similar STDP system (Diehl and Cook, 2015) on the original MNIST obtained 87.0% accuracy. On an 2,400 neuron network, our system achieved 91.78% accuracy while a 1,600 network (Diehl and Cook, 2015) achieved 91.9% accuracy. It is not surprising to see a slight deterioration in N-MNIST over MNIST due to noisy and more realistic input.

This is an empirical paper, and as such we do not prove that additional temporal information contained in spike timings is not present in the datasets. We do however, clearly show that the results point in this direction. In the first part of the paper, the comparable accuracy between the ANNs and state-of-art SNNs could lead to two possible conclusions: (1) No additional temporal information in the timing of spikes is available in the datasets, so an ANN can perform just as well, or (2) There is, but existing SNN methods do not make proper use of the additional temporal information. After all, research on ANNs is much more mature than that of SNNs, and ANNs are generally expected to perform better. These results are significant because of the reasons given as follows.

N-MNIST and N-Caltech101 have actually been used to assess many SNN algorithms. However, the fact that an ANN (such as the CNN used for image classification) which uses no additional temporal information contained in spike timings is on par with these SNNs shows that (1) These SNNs are either not using the additional temporal information, or (2) No such temporal information is available. In either case, the efficacy of these SNNs has not been proven. The implication of our finding is the below: with already state-of-the art or close to state-of-the art accuracies achieved by an ANN (specifically a standard CNN for image classification) based on collapsed neuromorphic datasets, if this is due to inherent lack of useful additional temporal information, such datasets cannot be used in SNNs or in general any machine learning algorithms hoping to leverage on spatio-temporal information in these datasets. If however, it is due to the fact that existing SNNs are found lacking in leveraging on the encoded spatio-temporal information, then would it not be more conclusive (and also satisfying) to develop better SNNs for datasets that standard ANNs could not do well in, and demonstrate some significant improvements rather than marginal ones in terms of accuracy? This marginal improvement would be problematic in justifying the efficacy of the newly developed SNN anyway, as it is always difficult to tease out the role of hyper-parameter tuning. Hence, in any case, while the paper aims to empirically show that there is little useful spatio-temporal information in such neuromorphic datasets, should the reader remains unconvinced, one should at the very least, bear in mind that there is little to be gained over the close to already state-of-art accuracies obtained from using standard CNNs.

One could imagine that if there is any useful additional temporal information contained in the timing of spikes, then collapsing the spike trains over its entire duration of all 3 saccades would have lost all of this information. We next train a standard CNN using this dataset obtaining an accuracy of 99.18% showing that there is not much change in the performance at all. This shows that changing time bins does not cause the performance to deteriorate. It also casts serious doubt on if there is any additional temporal information contained in spike timings in these neuromorphic datasets, hence requiring more studies (part two of the paper) in addressing this.

While we do not expect datasets derived from static images to have additional temporal information in the timing of spikes, we do expect recordings of movements to contain temporal information. Therefore we expect that in a dataset such as DvsGesture, ANNs cannot match the performance of SNNs as this dataset is expected to contain additional temporal information. Indeed, we note that this is correct—in an ANN identical to one that was used for N-MNIST, we obtain an accuracy of 71.01% which is far less than state-of-the-art SNN accuracy which is mostly greater than 95%. If, indeed, the results on N-MNIST and N-Caltech101 were because current SNNs were unable to extract additional temporal information in spike timings that is present in the dataset, then why does DvsGesture have a different result? Indeed, an SNN is able to extract the relevant temporal information, and perform far better than our ANN in classifying the DvsGesture dataset.

We initially approached N-MNIST to devise a STDP algorithm for classifying neuromorphic data, and as a result we implemented the first unsupervised SNN algorithm for N-MNIST. However, explorations with N-MNIST showed that its features encoded are not discriminative in time. These results are confirmed in N-Caltech101 as well. In this section, we detail why this result is important, and discuss the possible next steps. We pose several questions: (1) Why do we get these results? (2) Why do we need a neuromorphic dataset that is discriminative in the time domain? (3) What constitutes a neuromorphic dataset that can evaluate the temporal aspect of neuromorphic ability? If N-MNIST is not suitable, then what is? This is a very important question in neuromorphic engineering.

Why do we get these results? We get good results in the ANN (section 3) and rate-based SNN (section 5) due to the nature of N-MNIST. We sum up the spikes in an N-MNIST saccade in two ways (1) through collapsing the events in time as in section 3 or (2) by a relatively non-leaky integration of spikes in section 5. Using both methods, we note that after summation we retain all the information in N-MNIST (see Figure 2 for a few examples of collapsed images). This is possibly because of the static 2-dimensional nature of the underlying dataset (i.e., MNIST). Using the N-MNIST creation process of recording from the ATIS camera can at best reproduce the original MNIST dataset—there is no additional information over time. N-MNIST is less informative than MNIST, due to noise and gradations in the image introduced due to the moving camera. Noise is good, as the recordings from the camera make the dataset more realistic. Gradation in the image—i.e., high spike rate while recording certain parts of the image and low spike rates in other parts of the image—is an artifact introduced by the predefined and regular N-MNIST camera movements. Such gradations do occur in the real world. However, as our sensory neurons are able to detect and embody the statistics in the environment (Simoncelli and Olshausen, 2001; Geisler, 2008; Elder et al., 2016) the image gradations represented in biological neurons are not an artifact of biological image processing, but probably accurately reflect the statistics of the scene itself.

We get good results in the last experiment (section 7) due to an artifact in the N-MNIST dataset. The ATIS camera movements are clearly defined, regular, and all images are relatively similarly sized. Such regularity is not characteristic of retinal saccades, or any other sensory stimuli. Since we do not believe N-MNIST to encode discriminative features in time, we could then exploit such an artifact to do a rate-based classification, as we rightfully demonstrate in section 7. There are others who agree with our point of view on the limitations of datasets such as N-MNIST (for e.g., Sethi and Suri, 2019; Zhu et al., 2019; Deng et al., 2020; He et al., 2020; See et al., 2020), and He et al. (2020) shows similar results in a different paradigm (i.e., RNN vs. SNN) to further corroborate our point.

Why do we need a dataset that is discriminative in the time domain? The ability to use precise spike timings in calculations is a very useful property of SNNs, and we need more datasets that are able to evaluate this property. The spirit of neuromorphic engineering is not to just reproduce the methodology and computational mechanisms that deep learning already has, but to utilize additional characteristics of spiking neurons such as precise spike timings. We argue that given the event-based nature of the DVS camera, it is an ideal sensor platform to generate event datasets for benchmarking SNNs. However SNNs should not only be able to learn spike count/rate encoded information but also precise spike timing encoded information. As such, we hope to see more DVS datasets which encode information in precise spike timing, such as the DvsGesture. As seen in the introduction of this paper, there is a lot of biological evidence that precise spike times play an important role in neural computations. The brain works on spatiotemporal patterns. SNNs use spikes as their units of computation. STDP uses difference between spike times as its measure for learning. To highlight the utility of these computational mechanisms, we need datasets wherein features are encoded in individual spike times asynchronously.

In order to do well on a rate-based dataset, large time constants for synaptic traces are required to sum up over spikes. This necessarily results in slower reaction times. As we have stated in our introduction, one of the arguments by Thorpe for spike time coding in SNNs is that biological systems have short reaction times. Therefore, we do think that in a cognitive task that requires fast response time, spike time coding maybe more biologically plausible. The development of better SNN learning algorithms we believe is also largely driven by the quest for an algorithm that can learn the temporal information encoded in spike timing and its derivatives. Naturally, the dataset to assess such algorithms should then contain useful time information necessary for the classification task. We think audio and motion datasets would contain such temporal information, and learning algorithms sensitive to spike timing would have small time constants for their synaptic traces, leading to shorter reaction time as well.

Finally, our third and most important question is—what constitutes a neuromorphic dataset that can evaluate the temporal aspect of neuromorphic ability? The method of moving images or a vision sensor across static images in a Computer Vision dataset was one of the first attempts at creating a neuromorphic dataset. Although researchers have used datasets such as N-MNIST and N-Caltech101 for various purposes, we have seen that they do not have additional temporal information contained in spike timing necessary for their classification. What kind of dataset has this temporal information? We believe that DvsGesture does as it has recordings of dynamic movements—information that varies over time. Other useful candidates may be audio and video datasets. Audio and video are inherently spatiotemporal, and summing up temporal events over time will result in huge loss of information. These datasets also does not have one single peak in amplitude that is representative of all patterns. Over a short duration, audio and video do not make sense. On the contrary, audio and video events are dynamic, and events that unfold over a period of time lead to a holistic representation of the information, as described in George (2008).

There are several studies in speech classification where deep learning methods are applied to spectrograms which are treated like static images. This is indeed one interesting approach to audio classification, alongside other approaches using recurrent neural networks or the LSTM. An advantage of SNN (for instance one trained using the tempotron) over deep learning methods is its ability to predict the class as soon as there is enough discriminatory evidence, and not at the end of the input (Gutig and Sompolinsky, 2006). This is achieved even when the SNN is trained over the entire audio sequence duration. Another would be that when an ANN is trained using the multi-condition protocol (McLoughlin et al., 2015), the accuracy for clean data tends to suffer a little, while this is not the case for a SNN. Both these advantages are discussed in work still under review. He et al. (2020) show that datasets not derived from static images (i.e., DvsGesture) are more suitable for SNNs than RNNs. On the other hand, datasets such as N-MNIST do not show this advantage.

N-MNIST and N-Caltech101 and the datasets from which they were derived, i.e., MNIST and Caltech101, are inherently about image classification, and DvsGesture about action recognition. However, an image classification dataset can have information encoded not just in the spatial domain (Fiser and Aslin, 2002; George, 2008). Motion and action classification requires data changing over space and time. Although we are able to recognize a static image perfectly well, we are also able to generalize in a way that deep learning cannot—over different rotations, lighting conditions, sizes, and so on. This is possibly because we are exposed to a continuous stream of varying data (Simoncelli, 2003; Blake and Lee, 2005; Mazzoni et al., 2011; Faive and Koch, 2014; Keitel et al., 2017), and use time as a supervisor to understand and perform these generalizations (George, 2008). A visual dataset that embodies these principles may be suitable. Saccades in biological systems in the real world are over objects which may be moving or even if stationary, changing in perspective over time. In this case, collapsing over saccades will lose this time encoded information useful for cognitive functions, as additional information on precise spike timing is lost. Considering the changes along with the precise time information will lead to holistic representations not otherwise possible with static information. Clearly, N-MNIST and N-Caltech101 have information encoded in spatial-temporal domain, albeit the time domain encoding scheme being spike count based. Images can also be encoded in the temporal domain using precise spike timing, as in the case of latency coding (Mostafa, 2018; Comsa et al., 2020), for instance. Similarly, for a temporal dataset like speech, one can use a CNN to learn such a dataset whereby the input is encoded using an image generated from a spectrogram of the input word (Palaz and Collobert, 2015). Therefore the creation of a spatiotemporal dataset need not be limited to a particular task, but rather the manner in which the data is encoded.

## 9. Conclusion

In this paper, we address an important issue in neuromorphic computing by examining if datasets created from static images with the DVS-camera are discriminative over the time domain. We have focused on N-MNIST throughout the paper, but in the first experiment, show that N-Caltech101 follows the same trend. In the discussion, we have highlighted why it is important to have datasets that are discriminative in time. We also discuss what would be an appropriate dataset that tests the ability of SNNs to use precise spike timings in their computation.

In conclusion, spikes occurring over time is not just an alternate mechanism for representing static information, such as using the intensity of a pixel as the rate for a Poisson spike train. Brains have evolved to use computing mechanisms that are inherently suitable to represent and process information from a dynamic world, and even for a purely engineering purposes, we can utilize these processes. This paper, therefore highlights a need for further research into effective benchmarks that could test the temporal abilities of SNNs over earlier neural networks.

## Data Availability Statement

The complete results presented in the studies in section 5 are included in the article/Supplementary Material, further inquiries can be directed to the corresponding author/s.

## Author Contributions

YC and LI conceived the overall idea of the paper. Experiments were designed by YC and LI and shaped further through discussions between them. The paper was written by LI and edited by YC who improved the paper with useful additions. The experiments were conducted primarily by LI with some code written by YC. HL did the overall editing and contributed some interesting ideas. All authors contributed to the article and approved the submitted version.

## Conflict of Interest

YC was employed by the company Huawei Technologies. The remaining authors declare that the research was conducted in the absence of any commercial or financial relationships that could be construed as a potential conflict of interest.

## Footnotes

^1^https://rethinkresearch.biz/articles/intel-unveils-loihi-neuromorphic-chip

^2^https://github.com/gorchard/Matlab_AER_vision_functions/

^3^http://www.kaggle.com
