# Supplementary material for: Is Neuromorphic MNIST Neuromorphic? Analyzing the Discriminative Power of Neuromorphic Datasets in the Time Domain
Source: Front Neurosci. 2021 Mar 25;15:608567. doi: 10.3389/fnins.2021.608567 (PMC8027306; doi:10.3389/fnins.2021.608567)
Supplement: Supplementary file 1 [file Data_Sheet_1.PDF]

Supplementary Material

1 DETAILED RESULTS OF DSE1

Since the complete results of $DSE1$ are difficult to show in the main paper, we show these results in this section. The results of $DSE2$, however, are contained in the main paper itself.

This section shows in detail, all the results of *DSE1* for both N-MNIST and DVSGesture. The numbers indicate the test accuracy on the system for the particular combination of parameters. *DSE1* is a design space exploration of two parameters, EPSC current A_{xe} and learning rate η for O-SNN and three parameters, EPSC current A_{xe}, learning rate η and amplitude of threshold adaptation $\Delta\theta$ for T-SNN. For more details, refer to Section 5 of the main paper.

1.1 O-SNN

An X in any cell indicates that for this set of parameters, the classification could not occur because the spike frequency adaptation threshold is too high. The the system cannot produce spikes, and as a result, the system gets stuck in an infinite loop, trying to produce spikes.

DVSGesture

$\Delta\theta$ \ η	0.0005	0.005	0.05	0.5
0.01 mV	23.20 %	21.15 %	11.50 %	10.47 %
0.1 mV	36.96 %	46.41 %	37.78 %	53.18 %
1 mV	38.60 %	42.51 %	35.11 %	22.59 %

N-MNIST

$\Delta\theta$ \ η	0.0005	0.005	0.05	0.5
0.01 mV	X	X	X	X
0.1 mV	83.02 %	83.89 %	83.89 %	76.41 %
1 mV	58.05 %	82.99 %	79.68 %	77.51 %

Note that even though there are many X values in this experiment, the best results, i.e. 83.89% are present in this case.

1.2 T-SNN

An X in any cell indicates that for this set of parameters, the classification could not occur because for most patterns, the system did not fire any output spikes.

DVSGestures

$A_{xe} = 0.1$ nA

$\Delta\theta$ \ η	0.0005	0.005	0.05	0.5
0.01 mV	X	X	X	X
0.1 mV	X	X	X	X
1 mV	X	X	X	X

$A_{xe} = 0.3$ nA

$\Delta\theta$ \ η	0.0005	0.005	0.05	0.5
0.01 mV	52.57 %	53.39 %	32.65 %	X
0.1 mV	14.37 %	X	X	10.27 %
1 mV	X	X	X	X

$A_{xe} = 0.5$ nA

$\Delta\theta$ \ η	0.0005	0.005	0.05	0.5
0.01 mV	45.17 %	43.53 %	36.96 %	X
0.1 mV	X	X	55.44 %	25.05 %
1 mV	9.65 %	X	X	X

$A_{xe} = 0.7$ nA

$\Delta\theta$ \ η	0.0005	0.005	0.05	0.5
0.01 mV	59.11 %	40.25 %	29.98 %	12.53 %
0.1 mV	X	57.91 %	49.49 %	23.20 %
1 mV	X	X	X	X

N-MNIST

$A_{xe} = 1.0$ nA

$\Delta\theta$ \ η	0.0005	0.005	0.05	0.5
0.01 mV	75.51 %	76.13 %	75.30 %	X
0.1 mV	X	X	X	X
1 mV	X	X	34.39 %	X

$A_{xe} = 3.0$ nA

$\Delta\theta$ \ η	0.0005	0.005	0.05	0.5
0.01 mV	X	68.27 %	49.69 %	16.13 %
0.1 mV	X	59.99 %	38.84 %	9.74 %
1 mV	X	X	X	X

$A_{xe} = 5.0$ nA

$\Delta\theta$ \ η	0.0005	0.005	0.05	0.5
0.01 mV	61.32 %	55.16 %	29.20 %	10.66 %
0.1 mV	X	47.68 %	0.1523 %	11.35 %
1 mV	X	X	X	9.74 %

$A_{xe} = 7.0$ nA

$\Delta\theta$ \ η	0.0005	0.005	0.05	0.5
0.01 mV	51.62 %	45.21 %	49.07 %	11.94 %
0.1 mV	23.19 %	39.60 %	40.98 %	11.35 %
1 mV	12.04 %	19.22 %	X	X
